# Cadmium transport by mammalian ATP-binding cassette transporters

**DOI:** 10.1007/s10534-024-00582-5

**Published:** 2024-02-06

**Authors:** Frank Thévenod, Wing-Kee Lee

**Affiliations:** 1 Institute for Physiology, Pathophysiology and Toxicology & ZBAF, Witten/Herdecke University, 58453 Witten, Germany; 2 Physiology and Pathophysiology of Cells and Membranes, Medical School OWL, Bielefeld University, Morgenbreede 1, 33615 Bielefeld, Germany

**Keywords:** ABC transporters, Toxicity, Transition metal, Metal speciation, Membrane transport

## Abstract

Cellular responses to toxic metals depend on metal accessibility to intracellular targets, reaching interaction sites, and the intracellular metal concentration, which is mainly determined by uptake pathways, binding/sequestration and efflux pathways. ATP-binding cassette (ABC) transporters are ubiquitous in the human body—usually in epithelia—and are responsible for the transfer of indispensable physiological substrates (e.g. lipids and heme), protection against potentially toxic substances, maintenance of fluid composition, and excretion of metabolic waste products. Derailed regulation and gene variants of ABC transporters culminate in a wide array of pathophysiological disease states, such as oncogenic multidrug resistance or cystic fibrosis. Cadmium (Cd) has no known physiological role in mammalians and poses a health risk due to its release into the environment as a result of industrial activities, and eventually passes into the food chain. Epithelial cells, especially within the liver, lungs, gastrointestinal tract and kidneys, are particularly susceptible to the multifaceted effects of Cd because of the plethora of uptake pathways available. Pertinent to their broad substrate spectra, ABC transporters represent a major cellular efflux pathway for Cd and Cd complexes. In this review, we summarize current knowledge concerning transport of Cd and its complexes (mainly Cd bound to glutathione) by the ABC transporters ABCB1 (P-glycoprotein, MDR1), ABCB6, ABCC1 (multidrug resistance related protein 1, MRP1), ABCC7 (cystic fibrosis transmembrane regulator, CFTR), and ABCG2 (breast cancer related protein, BCRP). Potential detoxification strategies underlying ABC transporter-mediated efflux of Cd and Cd complexes are discussed.

## Introduction

Human exposure to cadmium (Cd) mainly arises from natural sources, such as volcanic eruptions, weathering and erosion, and river transport as well as from human activities, such as tobacco smoking, mining, smelting and refining of non-ferrous metals, fossil fuel combustion, incineration of municipal waste, manufacture of phosphate fertilizers, and recycling of Cd-plated steel scrap and electronic waste (WHO 2019). Mammalians either inhale Cd-containing particles (e.g. Cd-oxide), with cigarette smoking as the main source, or ingest Cd complexes with food and drinks. Cd is more concentrated in food items, such as shellfish, offal and crops (e.g. rice, soybeans or wheat), which accumulate Cd from contaminated soils and water. In the body, Cd forms high-affinity complexes with metalloproteins [e.g. metallothionein (MT)] and estrogen receptor, but it also interacts at low affinity with zinc-finger proteins, iron-binding proteins, and various low- and high-molecular weight plasma proteins (reviewed in Maret and Moulis 2013; Moulis 2010; Thévenod and Wolff 2016). Moreover, Cd forms complexes with a variety of organic molecules with relevance to biological systems (Carballo et al. 2013), including sugar residues, nucleobases (Sigel et al. 2013), amino acids, and peptides (Sovago and Varnagy 2013).

ATP-binding cassette (ABC) transporters comprise one of the largest and most ancient protein families expressed in living organisms. They operate as molecular machines by coupling ATP binding, hydrolysis, and phosphate release to the directional transport of a multitude of structurally diverse substrates across membranes (Thomas and Tampe 2020). Their substrates range from vitamins, steroids, lipids, and ions to peptides, proteins, polysaccharides, but also xenobiotics (Thomas and Tampe 2018). Because of their ubiquitous presence in nature and diverse physiological functions, mutations of ABC proteins cause various human diseases, e.g. cystic fibrosis, hypercholesterolemia, retinal degenerations, and lipid trafficking disorders (Moore et al. 2023). In addition, ABC transporters are responsible for multidrug resistance (MDR), which leads to antibiotic resistance in bacteria (Orelle et al. 2019) and cancer chemotherapy resistance (Robey et al. 2018). Interestingly, the plant genome encodes for more than 100 ABC transporters, largely exceeding that of other organisms. These ABC transporters are involved in many aspects of plant life, including development and survival (Kang et al. 2011). 

Because several ABC transporters are involved in detoxification and/or extrusion of environmental toxins and xenobiotics, they are often expressed in plasma membranes of cellular layers lining internal or external surfaces, such as epithelia and endothelia (Glavinas et al. 2004), to protect organs and organisms from the harmful effects of toxic compounds.

## Cadmium (Cd)

### Speciation

According to the International Union of Pure and Applied Chemistry (IUPAC), the chemical species of an element is the “*specific form of an element defined as to isotopic composition, electronic or oxidation state, and/or complex or molecular structure*” (McNaught and Wilkinson 1997). In the context of metals, these can be found in free ionic or bound states, with various donor ligands, and differing oxidation states. More often than not, metals are present in multiple species that are ultimately determined by the composition of the surrounding environment, for example, inorganic ions/ligands, other metals, organic compounds, temperature and pH. Metal speciation changes its chemico-physical properties and thus has large impact on how animals, plants and humans are affected by metals and metals mixtures.

Cd is a soft metal and contains two valence electrons in its outer shell therefore leading to +2 oxidation state (Cd^2+^) in compounds, as these two electrons are generally lost. It prefers forming stable complexes with soft donor atoms, such as S, N and O (Andersen 1984). In fact, Cd exhibits coordination numbers varying from three to eight in complexes with nucleobases, proteins, phosphoric groups, lipids, amino acids, sugars, vitamins, and thiols (Carballo et al. 2013), amongst others. In humans, Cd complexation with thiol groups [in cysteine and glutathione (GSH)] is of particular importance in its toxicology and pathophysiological effects. The multifaceted complexity of Cd speciation seems to have hampered systematic measurements of contaminated environmental sources as well as in human bodily fluids and tissues.

Several studies highlight the variability of Cd species present in atmospheric air, water and soils. In the atmosphere, Cd is found on particulate matter less than 2.5 µm in diameter (PM2.5), particularly in urban areas (Kermani et al. 2021; Li et al. 2022), primarily resulting from cigarette smoke and tyre wear dust. Industrial activities, such as incineration and metal production, release Cd in inorganic compounds (CdCl_2_, CdS, CdSO_4_), or bioinorganic complexes when organic material is present (Crea et al. 2013). In natural freshwater, ionic cadmium (Cd^2+^) represents approximately half of Cd species whereas in seawater, cadmium chloride complexes (CdCl^+^, CdCl_2_, CdCl_3_^−^) dominate (Crea et al. 2013). Typical soils comprise of minerals, organic matter, water and air yet this varies greatly across different environments. Trace element speciation is usually affected by soil pH, minerals and organic matter but also by time/aging, temperature and movement, and will determine the available amount of soluble and exchangeable Cd. Cd stabilization will decrease the exchangeable form and mobility of Cd and in turn decrease its bioavailability, which has important consequences on its uptake into plants and other organisms. For example, increased chloride concentration results in increased Cd bioavailability to plants (Weggler et al. 2004), and low soil pH (Tian et al. 2022), or soil flooding and drainage (Yan et al. 2021) increases exchangeable Cd. Due to the large variabilities in soil composition and thus their physicochemical properties, it is difficult to reconcile all observations reported and the reader is referred to the following reviews: (Crea et al. 2013; Peana et al. 2021; Li et al. 2021).

**Fig. 1:**
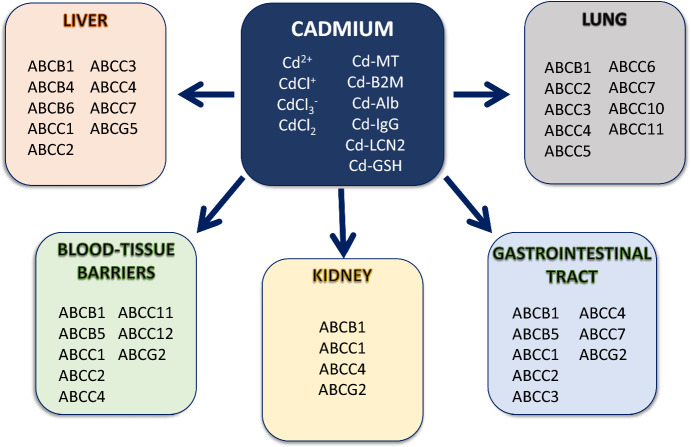
Cadmium speciation, target organs and ABC transporter expression. Cadmium is present in the environment and in living organisms in different forms. Cadmium chloride (CdCl_2_) dissociates mostly into chloro-complexes. Once ingested, cadmium can bind to various ligands, including metallothionein (MT), beta-2-microglobulin (B2M), albumin (Alb), immunoglobulin G (IgG), lipocalin-2 (LCN2), and glutathione (GSH). Organs and tissues affected by cadmium toxicity and their ABC transporter expression are depicted. Based on current knowledge, it is not possible to allocate cadmium species as substrates to each specific ABC transporter. Please refer to the main text for details

In humans, knowledge about Cd speciation has primarily been derived from measurements made in blood plasma. A number of reports have demonstrated complexation of Cd in vitro or in vivo with organic molecules: MT, a zinc-binding protein, the antioxidant cysteine-containing tripeptide GSH, transferrin (Harris and Madsen 1988), cysteine, apolipoprotein A1 (Li et al. 2020), β2-microglobulin, albumin, lipocalin-2, and immunoglobulin G (Fels et al. 2019) (Fig. 1). Intriguingly, Cd forms complexes with bicarbonate and is transported by the HCO_3_^−^/Cl^−^ exchanger (SLC4A1) present in erythrocyte membranes in an anion exchange inhibitor DIDS (4,4′-diisothiocyano-2,2′-stilbenedisulfonic acid)-sensitive manner (Lou et al. 1991), indicating bioinorganic chemistry also contributes to Cd effects in the blood and beyond.

It is important to highlight the impact of organic molecules. In an exemplary study, Nair and Robinson predicted 97% Cd-chloro complexes and 3% free Cd in blood plasma of bivalves using an inorganic fluid composition. When a Cd-binding histidine rich glycoprotein was included, 86.8% was protein bound, 11.6% was present as Cd-chloro complexes and 1.3% as Cd (Nair and Robinson 2000). Despite the complex and dynamic compositions of human bodily fluids, predictions made with key metal-binding molecules in the fluid composition would give crucial information regarding the major Cd species and complexes.

Initial observations regarding Cd speciation and uptake into mammalian cells (renal proximal tubule LLC-PK1) demonstrated CdSO_4_ salts were more toxic than CdCl_2_ salts in serum- and bovine serum albumin (BSA)-free medium whereas no difference in uptake was observed between the compounds in serum or BSA-containing medium. Furthermore, uptake into cells was dependent on protein uptake rates, therefore increased uptake was achieved in BSA-medium (~ 26%) in contrast to only ~ 17% uptake in serum-medium (Barrouillet et al. 2001). Interestingly, pH did not affect Cd cytotoxicity even though higher pH levels result in increased Cd toxicity in bacteria (Worden et al. 2009), further reiterating the need for advanced studies for analysis of Cd speciation in different biological systems.

### Toxicity

#### Acute toxicity

The symptoms of acute high-dose Cd intoxication depend on the route of ingestion (reviewed in (Thévenod and Lee 2013b)). Inhalation of fumes in an industrial setting affects the lungs and results in acute pneumonitis, pulmonary edema with respiratory failure, and possibly death (Yates and Goldman 1990; Ellenhorn and Barceloux 1996; WHO 1992). Ingestion of high doses of Cd is rare and either accidental or intentional. Acute high-dose Cd intoxication mainly damages the liver, which is the cause of death (Buckler et al. 1986; Bernard and Lauwerys 1986; WHO 1992). Cardiovascular collapse may also occur with ensuing acute organ failure (kidneys, heart, lungs, liver), which may also cause death (Buckler et al. 1986; Bernard and Lauwerys 1986; Ellenhorn and Barceloux 1996; WHO 1992). Acute Cd intoxication may also induce necrosis of the testes and other reproductive organs and result in infertility (Kumar and Sharma 2019; Siu et al. 2009). At the molecular level, oxidative stress plays a major role in cellular damage of these organs (Liu et al. 2008). Disruption of the cellular GSH system, inflammatory processes in the liver, and the Fenton reaction driven by iron mainly contribute to reactive oxygen species (ROS) formation in acute hepatotoxicity induced by Cd (reviewed in (Liu et al. 2009)). Although no similar data are available for lungs, analogous molecular mechanisms may be responsible for acute pulmonary toxicity (Xiong et al. 2019).

#### Chronic toxicity

The kidneys and liver combined contain ~ 85% of the Cd body burden, and more than 60% was found in the kidneys in the age range of 30–60 years (Salmela et al. 1983). The accumulation of Cd in the kidney and its very long biological half-life may explain the increased susceptibility of the kidney to the toxic effects of Cd (Buchet et al. 1990; Jarup and Alfven 2004). This may also account for increased incidence of chronic kidney disease and end-stage renal failure in populations with chronic low Cd exposure (CLCE) (Satarug 2018; Hellstrom et al. 2001). Damage and dysfunction of many other organs are also induced by CLCE, such as the liver, respiratory system, bone, cardiovascular, reproductive and nervous system, endocrine glands, or hematopoiesis (Thévenod and Lee 2013b) (Fig. 1), which increases the risk of mortality (reviewed in (Larsson and Wolk 2016)).

Teratogenic and epigenetic effects of environmental Cd in humans and acute or chronic exposure (mainly subcutaneous or intraperitoneal injection of CdCl_2_) in experimental animals have been recently reviewed (Jacobo-Estrada et al. 2017; Geng and Wang 2019; Lawless et al. 2023). Exposure to Cd exposure during human pregnancy induces damage to the mothers (e.g. preeclampsia with kidney damage and secondary effects, such as imbalance of hormonal status, ion and fluid homeostasis, or bone calcification). Cd also causes placental toxicity with oxidative stress and alterations of copper and zinc homeostasis in rodents. Abnormal fetal development also occurs (e.g. growth deficiencies; alterations of central nervous system, kidneys, liver and bone; increased oxidative stress/impairment of anti-oxidative system; altered homeostasis of iron, zinc, copper and calcium) (Jacobo-Estrada et al. 2017). The coexistence of placental and embryonic toxicity of Cd in different species suggests similar toxicological mechanisms. Hence, comparable epigenetic alterations within cord blood, placenta, and fetal tissue upon Cd exposure may lead to pregnancy complications, such as preeclampsia and poor fetal growth. These biological disruptions viewed in the fetal and neonatal stages may lead to premature death of the fetus, persist into childhood, but may also result in various systemic diseases during adulthood, such as hypertension, obesity, and diabetes (Geng and Wang 2019). Furthermore, epigenetic alterations within germ cells pose multigenerational adverse effects on the reproductive system. Interestingly, sex-specific effects on epigenetic mechanisms also occur, such as gene hypermethylation in male offspring and hypomethylation in females, as demonstrated in humans exposed to environmental Cd (reviewed in (Lawless et al. 2023)).

Cd exposure has been linked to tumors of multiple organs/tissues in humans and animals (reviewed in Nawrot et al. 2010; Huff et al. 2007; WHO 2019). An association between Cd exposure and the occurrence of cancer in the lung (Nawrot et al. 2015; Adams et al. 2012) has led the International Agency for Research on Cancer (IARC) to classify Cd as a human carcinogen (WHO 2019; IARC 1993). Cd exposure in humans is also associated with increased incidences of renal cancers (Il’yasova and Schwartz 2005; DFG 2006; reviewed in (WHO 2019)). The evidence for Cd-associated development of prostate cancer in humans is, however, less consistent (WHO 2019; Verougstraete et al. 2003). Case–control studies suggest that other cancer sites, such as the bladder, the mammary gland, and the endometrium may show increased risks associated with dietary or respiratory Cd exposure (Akesson et al. 2008; McElroy et al. 2006; Kellen et al. 2007; reviewed in (WHO 2019)). Moreover, Cd can induce tumors of the lung, prostate, testis, pancreas, adrenals, liver, kidney, pituitary, and hematopoietic system in mice, rats, or hamsters (reviewed in (Goyer et al. 2004; Huff et al. 2007; Waalkes 2003)).

Current evidence indicates that Cd is not directly genotoxic. Rather multiple indirect mechanisms underlie CLCE-induced carcinogenesis, such as alterations in gene expression patterns, interference with the cellular DNA damage repair systems, and oxidative stress (Hartwig 2013; Chen et al. 2019; Zhu and Costa 2020). Evidence for the latter mechanism is strong (reviewed in (Hartwig 2013; Nair et al. 2013; Nemmiche 2017)), although not uncontested (Liu et al. 2009). Significant work emphasizes the contribution of epigenetic mechanisms to CLCE-mediated carcinogenesis (Waalkes 2003; Arita and Costa 2009; Martinez-Zamudio and Ha 2011; Humphries et al. 2016; Chen et al. 2019; Zhu and Costa 2020; Zhao et al. 2022) or interference with pro-apoptotic mechanisms (Thévenod and Lee 2013a; b; 2015; Chen and Costa 2017).

At the molecular and cellular level Cd interferes with redox and calcium signaling, and essential transition metal ion homeostasis, such as iron, copper, zinc and manganese, which has been described in detail elsewhere (Thévenod and Lee 2013a; Thévenod 2009; Moulis 2010).

## ABC transporters

### General structure and function

The evolutionary-conserved ABC transporter superfamily is a large group of transmembrane (TM) transporters that utilize energy to translocate various substrates across membranes. Functional ABC transporters typically consist of at least two TM domains (TMDs) and two nucleotide binding domains (NBDs) (Ambudkar et al. 2003), either synthesized from a single polypeptide chain for a full transporter or from two separate chains with each forming a half transporter harboring one TMD and NBD that come together to form a full transporter (Baril et al. 2023; Alam and Locher 2023).

In the resting, pre-transport or apo-state, ABC transporters are typically found in a “triangular” inward-facing conformation where the bundles are in close proximity on the side where substrates are expulsed and the NBDs on the other side of the lipid bilayer are separated by up to approximately 40 Å. The substrate binding pocket is facing inward and may be open to both cytoplasm and inner leaflet of the lipid bilayer, as in the case of ABCB1/Pgp (Chen et al. 2001; Aller et al. 2009), but usually not to the extracellular compartment or the outer leaflet.

With a few notable exceptions, including the ion transporter ABCC7/CFTR and ion channel regulators ABCB8, ABCC8, ABCC9, ABC transporters function as unidirectional exporters of multiple substrates harnessing the energy from ATP hydrolysis to fuel conformational changes and substrate transport against energetically unfavorable concentration gradients. A diverse array of endogenous and physiological substrates is recognized and translocated, contributing to homeostasis, transfer between compartments, cellular and tissue protection, and excretion of excess or unwanted compounds. Exogenous substrates and xenobiotics are also translocated by some members of the ABC transporter family, which constitute the group of multidrug resistance transporters, of which ABCB1/Pgp, ABCC1/MRP1 and ABCG2/BCRP have the most significance. Together, they prevent passage of potentially toxic substances to protect susceptible tissues, such as the brain and testis (Glavinas et al. 2004; Leslie et al. 2005).

### Mechanisms of substrate transfer

Movement of substrates from one side of the membrane to another necessitates a coordinated sequence of events. Binding of the substrate(s) in the substrate binding pocket and ATP hydrolysis in the NBDs induce a conformational change and move the substrate from high-affinity to low-affinity binding, from where the substrate is released into the surrounding space, facilitated by ATP hydrolysis or by the law of mass action, and the transporter returns to its pre-transport state. Dynamic structural changes require strict choreography of the TMDs and the NBDs as well as maintaining its tertiary structure during these changes. Recent evidence from molecular dynamics simulations support an increasing role of TMD-connecting loops and linker regions in initiation and execution of the so-called catalytic or transport cycle through recognition sequences and elastic spring-like properties (Zolnerciks et al. 2014; Khunweeraphong et al. 2017; Dehghani-Ghahnaviyeh et al. 2021; Locher 2016).

Several models have been proposed for the exact mechanism of substrate recognition and transfer (Locher 2016). The simplest model describes substrate access from the aqueous phase, recognition and binding in the inward-facing confirmation and ATP hydrolysis-driven large structural change to the outward-facing conformation (van Meer et al. 2006). Further concepts describe a “vacuum cleaner” model wherein substrates partition in the lipid bilayer and access the binding pocket from the hydrophobic portion of the membrane (Chen et al. 2001; Qu and Sharom 2002), or the “flippase” model wherein substrates partitioned in the lipid bilayer are taken up through the cytoplasmic leaflet and “flipped” to the other membrane side (Eckford and Sharom 2005). Finally, it has been proposed that ABC transporters continuously cycle and substrates stochastically access the binding pocket during the inward-facing confirmation phase (Eytan 2005; Rauch 2011).

Since ABC transporters are largely found in lipid rafts within these membranes, it can be appreciated that lipid composition plays an integral role in their functionalization. Indeed, depletion of cholesterol and disruption of lipid rafts lead to loss of transport activity or reduction in ATP hydrolysis (reviewed in (Sharom 2014; Klappe et al. 2009; Hendrich and Michalak 2003; Lee and Kolesnick 2017)).

### Tissue expression and specific functions

Ubiquitously expressed ABC transporters provide essential functions in phospholipid and sterol homeostasis (e.g. ABCA1, ABCA7), mitochondrial function (ABCB8, ABCB10), or fatty acid transport (e.g. ABCD3). For further details, the reader is referred to recent reviews (Alam and Locher 2023; Thomas and Tampe 2018; 2020) and Fig. 1. Below, specific expression and function in major epithelia are described.

#### Liver

The major functions of the liver are metabolism, detoxification, excretion, storage and synthesis of metabolic substances. Together with several solute carriers, the luminal canalicular membrane has the capacity to transport organic anions (ABCC2/MRP2, ABCG2/BCRP), bile salts and acids (ABCC2/MRP2, ABCB11/BSEP [bile salt export pump]), the haemoglobin degradation product bilirubin (ABCC2/MRP2), organic cations (ABCB1/Pgp), and cholesterol (ABCG5/MOAT-C, ABCG8) that make up the major components of bile (Kroll et al. 2021; International Transporter et al. 2010) (Fig. 1). At the hepatocyte basolateral membrane, the presence of ABCC4/MOAT-B (bile acids and salts, GSH), ABCB4 (phospholipids), ABCC3/MRP3/cMOAT2, ABCC4/MOAT-B, (both drugs and metabolites), and ABCC6 (ATP) permit extrusion of substrates into the extracellular compartment and diffusion into the bloodstream. Lastly, ABCC7/CFTR mediates chloride transport in the bile duct membrane to maintain fluid homeostasis.

#### Respiratory tract and lung

The upper respiratory tract is continuously exposed to the harsh external environment through inhaled air. Accordingly, the luminal membrane of bronchial epithelial cells harbors multiple ABC transporters involved in detoxification and drug resistance (ABCB1/Pgp, ABCC2-6, ABCC10-11) (Chai et al. 2017) (Fig. 1). ABCC1/MRP1 is found in the basolateral membrane. ABCB1/Pgp also protects the airway epithelium from organic cations and the ABCC family members extrude organic anions. Creating an osmotic pressure through chloride transport, ABCC7/CFTR is responsible for increasing fluidity of viscous mucus secretions (van der Deen et al. 2005). The role of ABCC4/MOAT-B is not clear; it has been postulated to interact with ABCC7/CFTR to modulate cAMP signaling and potentiate ABCC7/CFTR activity (Nguyen et al. 2021).

#### Gastrointestinal tract

After mechanical, chemical and enzymatic breakdown of ingested food in the upper digestive tract, the small intestine is the major reabsorptive site for the transfer of essential energy-harboring substances from the lumen to the blood where they are further transported and processed for storage in the liver, fat and muscle. In addition, the intestine has a secretory and excretory function. Apical ABCB1/Pgp, ABCC2/MRP2, and ABCG2/BCRP in the enterocytes remove unwanted substances into the intestinal lumen where they can be excreted from the human body (Dietrich et al. 2003; Mutch et al. 2004) (see Fig. 1). Basolateral ABCC1/MRP1, ABCC3/c-MOAT2, ABCC4/MOAT-B, and ABCB5/MOAT-C move substrates into the blood. Similar to the aforementioned epithelia, ABCC7/CFTR is responsible for cAMP-dependent chloride secretion and fluidity of secretions (reviewed in De Lisle and Borowitz 2013).

#### Kidney

Blood filtration, detoxification, excretion, salt and water homeostasis, and reabsorption are the main functions of the kidneys executed by the functional unit, the nephron. In the proximal tubule (PT) of the nephron, ABCB1/Pgp extrudes organic cations whereas ABCC2/MRP2, ABCC4/MOAT-B and ABCG2/BCRP excrete organic anions into the tubular lumen for removal via the urine (Masereeuw and Russel 2012; Torres et al. 2021) (Fig. 1). All are expressed in the luminal membrane. Movement of these substances from the blood is mediated by members of the solute carrier (SLC22) family (organic anion and cation transporters) (Nigam 2018) in the basolateral membrane. Other ABC transporters expressed along the nephron after the PT have a less significant role in the handling of organic substances (Masereeuw and Russel 2012; Torres et al. 2021; Berg et al. 2021).

#### Blood-tissue barriers

To sustain life, neurons and glial cells of the brain and germ cells in reproductive tissues need to be safeguarded against potentially damaging agents. In the blood-–brain-barrier, the endothelial cells in the blood vessels use luminal ABCB1/Pgp, ABCG2/BCRP, ABCC4/MOAT-B, and ABCB5/MOAT-C to protect the brain (Miller 2010). With the exception of ABCB5/MOAT-C, the same transporters are expressed in the blood-retina-barrier (Chapy et al. 2016; Tagami et al. 2009). The blood-testis-barrier is formed by Sertoli cells in the seminiferous tubules where sperm production takes place. ABCB1/Pgp, ABCC1/MRP1, ABCG2/BCRP, ABCC11, ABCC12 in the Sertoli cells form the protective barrier for developing spermatozoa (Su et al. 2011; Miller and Cherrington 2018). In contrast, ABCB1/Pgp, ABCC1/MRP1, ABCC2, and ABCG2 expressed in placental syncytiotrophoblasts serve to shield the developing fetus from damaging agents (Yamashita and Markert 2021) (Fig. 1).

### Subcellular distribution

Generally, ABC transporters are found in the luminal membrane of polarized epithelial cells where they execute vectorial substrate transport into the extracellular space. There are some exceptions to the rule, such as ABCC1/MRP1 in the basolateral membrane. In some cells and under some pathophysiological states, ABC transporters are also found in intracellular membranes, such as trafficking vesicles, mitochondria, peroxisomes, Golgi or nuclei, depending on their substrate specificity and/or function. Their orientation within intracellular membranes is highly likely determined by the presence of the NBDs in the cytosolic compartment where ATP is in abundance. This has been experimentally evidenced for ABCB1/Pgp in acidic vesicles (Shapiro et al. 1998; Yamagishi et al. 2013) in addition to ABCC1/MRP1 (Jungsuwadee et al. 2009) and ABCB7 (Pearson and Cowan 2021) in mitochondria (reviewed in (Schaedler et al. 2015). Without organelle-specific localization sequences (in contrast to mitochondria-targeted ABC transporters), how ABC transporters reach these unintended sites of expression is currently not known though cellular membrane dynamics, such as microvesicle biogenesis, endo-/exocytosis, interorganellar communication or membrane lipid changes, are expected to be major contributing factors.

## ABC transporters and Cd

### ABCB1/Pgp

ABCB1/Pgp was first identified in drug-resistant cell lines, though subsequent studies ascertained it is, in fact, expressed physiologically, and not only under pathological conditions (Fojo et al. 1987; Thiebaut et al. 1987). Its expression is under transcriptional regulation (Scotto 2003; Lee et al. 2013) and subject to modifications by single nucleotide polymorphisms (SNPs) (Sauna et al. 2007; Wolking et al. 2015). ABCB1/Pgp is predominantly found at important epithelial linings, sites of demarcation between “internal” and “external” environments in the body, as well as in capillary endothelial cells at the blood-brain and blood-testis barriers. Epithelial cells lining the gut, liver, kidney, pancreas in addition to endothelial cells in blood-tissue barriers express high levels of ABCB1/Pgp in a highly-polarized fashion directing expulsion of physiological metabolites and xenobiotic compounds into a secretory duct or luminal system for excretion from the body (Thiebaut et al. 1987) or into blood, protecting tissues from damage (Fromm 2004).

Plasma membrane ABCB1/Pgp can transport a wide range of structurally-unrelated substrates directed towards the extracellular compartment: hydrophobic, amphiphilic, neutral or cationic compounds ranging from 300 to 4000 Da (Ambudkar et al. 2003; Chen and Simon 2000; Shapiro and Ling 1998; Alvarez et al. 1995; Aller et al. 2009). ABCB1/Pgp is also a lipid translocase with broad specificity (van Helvoort et al. 1996), extruding sphingomyelin (SM), glucosylceramide (GluCer), phosphatidylcholine (PC) and phosphatidylethanolamine (PE) (Eckford and Sharom 2005; Lee et al. 2011; Lee and Kolesnick 2017).

ABCB1/Pgp is found strongly expressed in intracellular compartments. Numerous studies locate functional ABCB1/Pgp to an acidic vesicular pool (Gervasoni et al. 1991; Shapiro et al. 1998; Yamagishi et al. 2013; Crivellato et al. 1999) where it sequesters cytosolic drugs and is sensitive to pharmacological inhibition (Shapiro et al. 1998; Crivellato et al. 1999; Yamagishi et al. 2013).

#### Cd and ABCB1/Pgp

ABCB1/Pgp is sensitive to stress signaling pathways. Upregulation of ABCB1/Pgp upon Cd exposure has been well-evidenced starting from the first observation by the Gottesman group (Chin et al. 1990). Since then, several transcription factors activated by Cd to govern ABCB1/Pgp expression have been identified, such as nuclear factor-kappa B (Thévenod et al. 2000), β-catenin/T-cell factor 4 (TCF4) (Chakraborty et al. 2010), paired-like homeodomain transcription factor 2 (PITX2) (Lee and Thévenod 2019), and c-myc (Thévenod et al. 2007), in addition to ABCB1/Pgp protein stabilization through heat shock protein 90 (Bertram et al. 1996).

Enhanced cell survival and decreased apoptotic cell death ensued elevated ABCB1/Pgp levels by Cd. Metal detoxification mediated by ABCB1/Pgp and other ABC transporters occurs in various models (e.g. (Callahan and Beverley 1991)), yet the underlying mechanism was not understood. To this end, it was hypothesized that Cd or Cd complexes would be effluxed by ABC transporters and hence remove toxic Cd to prevent cell death occurrence. In renal cell lines stably overexpressing ABCB1/Pgp, there are conflicting data alluding to the transport of Cd by ABCB1/Pgp. Sakata and colleagues used the porcine LLC-PK1 cell line transfected with human ABCB1/Pgp (LLC-GA5-COL150). Initial studies demonstrated enhanced accumulation of ^109^CdCl_2_ by up to twofold in LLC-PK1, LLC-GA5-COL150 and OK cells after preincubation with the functional ABCB1/Pgp antibody UIC2 or pharmacological ABCB1/Pgp inhibitors. Unexpectedly, co-incubation of inhibitors and ^109^CdCl_2_ did not result in ^109^CdCl_2_ accumulation in LLC-PK1 cells despite use of a 100-fold excess of the competitive inhibitor verapamil (100 µM versus 1 µM ^109^CdCl_2_) (Endo et al. 2002). In a further study using permeable supports, basolateral-to-apical transport of ^109^CdCl_2_ was augmented in LLC-GA5-COL150 compared to parental cells and was attenuated by the ABCB1/Pgp inhibitor cyclosporin A or UIC2 (Kimura et al. 2005). Both studies suggest Cd transport by ABCB1/Pgp though other effects of the modulators used cannot be ruled out.

In our own studies using MDCK cells stably overexpressing human ABCB1/Pgp or by increasing ABCB1/Pgp by constitutively active β-catenin, no inhibitory effect of UIC2, cyclosporin A and valspodar/PSC833 on ^109^CdCl_2_ efflux after 15–30 min were observed despite accumulation of ^109^CdCl_2_ into cytosolic and membrane fractions and reversal of ABCB1/Pgp-mediated cadmium resistance by valspodar/PSC833 in cell viability assays (Lee et al. 2011). Rather, the pro-apoptotic sphingolipid ceramide, and its glycosylated form glucosylceramide, were effluxed ~ twofold more by ABCB1/Pgp-MDCK compared to MDCK cells, and abolished by valspodar/PSC833 (Lee et al. 2011), which does not impact cellular ceramide levels per se (Dahdouh et al. 2014). This is in line with ABCB1/Pgp’s function as a lipid translocase (van Helvoort et al. 1996). Discrepancies between the two sets of studies could be a result of different cell lines as well as differences in experimental procedures, such as pre-incubation versus co-incubation or washing cells to remove cell surface bound Cd. Examining the chemical nature of ABCB1/Pgp substrates, metal ions appear to be unlikely candidates because of their hydrophilicity and inorganic chemistry. Electrophysiological studies using the patch clamp method and substrate cavity mutation studies would provide definitive evidence for Cd transport by ABCB1/Pgp.

### ABCB6

The ABCB6 half-transporter has several physiological roles in various tissues. Research has largely focussed on its capability to transport porphyrins (for e.g. heme synthesis) in multiple cell types (Krishnamurthy et al. 2006; Kim et al. 2022; Fukuda et al. 2016), its expression as the Langereis (Lan) blood group antigen on red blood cells (Helias et al. 2012), and its role in cell survival, primarily in cancerous cells derived from the liver (Zhang et al. 2020; Polireddy et al. 2011) and blood (Yin et al. 2023; Lynch et al. 2009). Emerging roles for ABCB6 have been linked to iron and iron-dependent cell death (ferroptosis) (Yin et al. 2023; Zhang et al. 2020) as well as offering protection against drugs (Minami et al. 2014; Murakami et al. 2020) and oxidative stress (Lynch et al. 2009).

Similar to other ABC transporters, ABCB6 can transport a wide range of substrates, including porphyrin and GSH (Polireddy et al. 2012). Substrate binding and substrate translocation seem to take place in two distinct cavities separated by a loop “plug”, serving as a restrictive barrier between an open cytoplasmic-accessible cavity-1, wherein substrates bind, and closed cavity-2, wherefrom substrates are translocated to the opposite compartment. Hydrophobic and basic amino acids line cavity-1 creating a hydrophobic and positively-charged environment that is favourable for hydrophobic and negatively-charged molecules (Song et al. 2021; Wang et al. 2020). In contrast to ABCB1/Pgp, the conformation of the TMD helices in ABCB6 prevents substrate uptake through the lipid membrane therefore ABCB6 recruits its substrates via the cytoplasmic side (Wang et al. 2020).

GSH is a tripeptide antioxidant particularly important in detoxification and cellular stress prevention, for example, in metal exposures. GSH, in addition to its oxidized form glutathione disulfide (GSSG) and GSH conjugates, is transported by ABCC1/MRP1, ABCC7/CFTR, and ABCG2 (Brechbuhl et al. 2010) (and possibly ABCC2 and ABCC4; see “Other ABC transporters”). Hence, it was hypothesized GSH is also a substrate for ABCB6 based on similar substrate binding pocket characteristics to ABCC1/MRP1. Wang et al. demonstrated direct GSH binding to ABCB6 using bio-layer interferometry, yet ATPase activity was not altered by GSH alone. Rather, porphyrin binding and consequent ATPase activity was strongly enhanced in the presence of GSH (Wang et al. 2020). In contrast, Song et al. observed a ~ twofold increase in ATPase activity by GSH, which was abolished by mutation of substrate binding amino acids S322A and T323A (Song et al. 2021).

Functional ABCB6 was originally located to the outer mitochondrial and plasma membranes in human leukemia and glioblastoma cell lines (Krishnamurthy et al. 2006; Paterson et al. 2007). More intricate subcellular fractionation using additional organelle markers revealed ABCB6 in integrin (for plasma membrane), complex III (for mitochondria) and LAMP1 (for lysosomes) positive fractions.

Conflicting discussions regarding transporter orientation has led to further investigation of the subcellular localization of ABCB6 in various models. Endogenous ABCB6 was found largely in apical early and late endosomes of intestinal cells in *C. elegans* (Kim et al. 2018), in melanosomes and lysosomes in an MNT-1 melanocyte cell line (Bergam et al. 2018), in plasma membranes of mature erythrocytes and in plasma membrane, exosomes and endosomes in reticulocytes (Kiss et al. 2012). Overexpression of rat ABCB6 in the human colon adenocarcinoma line LoVo resulted in labeling of endo-/lysosomes (Jalil et al. 2008) and overexpressed human ABCB6 in COS7 cells were localized to the ER and Golgi (Tsuchida et al. 2008), and to the endo-lysosomal compartment in SNB-19 glioblastoma cells (Rakvacs et al. 2019). Transient overexpression has come under scrutiny since it is not known whether the reported organelles are the final destination for ABCB6 or whether overactive expression leads to a backlog in the synthesis pathway, resulting in false positive signals for ABCB6.

#### Cd and ABCB6

With sequence homology to heavy metal tolerance factor 1 (HMT1) found in *Caenorhabditis elegans* (Schwartz et al. 2010) and other invertebrates, ABCB6 is considered as its human ortholog. Analogous to stress-sensitive ABC transporters, ABCB6 acts protectively against various stressors like peroxide and cyanide (Lynch et al. 2009), drugs (Minami et al. 2014) as well as selected metals (arsenite and Cd but not arsenate or copper) (Rakvacs et al. 2019; Kim et al. 2018). Multiple mechanisms appear to be involved in conferring protection. The simplest mechanism is accumulation of Cd and/or Cd complexes into organelles or extrusion across the plasma membrane to the extracellular side. Indeed, Cd was found in vacuoles when SpHMT1/ABCB6 was expressed in the fungus *Schizosaccharomyces pombe*, confirming previous observations of vacuolar SpHMT1/ABCB6 expression (Ortiz et al. 1995). Furthermore, SpHMT1/ABCB6 could rescue Cd toxicity in SpHMT1/ABCB6-deficient strains, wherein vacuolar Cd was diminished (Rakvacs et al. 2019). Intriguingly, ABCB6 overexpression conferred tolerance to Cd in glioblastoma cells, yet not in HeLa cells, and ABCB6 ATPase activity was not stimulated by Cd-GSH complexes (Rakvacs et al. 2019). Additional studies are required to ascertain the speciation of Cd that is transported by ABCB6 into endo-/lysosomes.

Localization of ABCB6 to endosomal recycling vesicles led to the hypothesis that it could take up potentially toxic by-products of Cd-heme interactions and extrude them from the cell (Kim et al. 2018). Finally, genetic manipulation of ABCB6 revealed positive regulation of expression and activity of catalase, a heme-containing enzyme that catalyzes the decomposition of hydrogen peroxide (Baker et al. 2023), suggesting increased availability of heme—as a direct result of ABCB6-mediated porphyrin transport—stabilizes catalase and thus provides augmented protection against oxidative stress (Lynch et al. 2009). This mechanism could be implicated in Cd toxicity, in which oxidative stress partly through inhibition of catalase is elicited (Lee et al. 2024; Probst et al. 2021).

### ABCC1/MRP1 (MRP1/multidrug resistance-associated protein 1)

The multidrug resistance-associated proteins are members of the C subfamily of ABC transporters. The subfamily ABCC contains thirteen members and nine of these transporters are referred to as the multidrug resistance proteins (MRPs). The MRP proteins are known to be involved in ion transport, toxin secretion, and signal transduction (Borst et al. 2000; Vore 2023), yet the physiological functions of many of them require further investigation. Some MRPs function as organic anion exporters and appear to have broad and partially overlapping substrate specificity. MRP1 encoded by ABCC1 was originally discovered as a cause of MDR in tumor cells (Cole et al. 1992), reviewed in (Cole 2014b). Although their degree of sequence homology is modest, the drug resistance pattern of ABCC1/MRP1 is much like that of ABCB1/Pgp and includes doxorubicin, daunorubicin, vincristine, colchicine and several other compounds (Xiao et al. 2021). However, it is now clear that ABCC1/MRP1 serves a broader role than simply mediating the ATP-dependent efflux of drugs from cells. The physiological substrate profile of ABCC1/MRP1 differs significantly from that of ABCB1/Pgp (Sharom 2011; Cole 2014a). While ABCB1/Pgp substrates are neutral or cationic lipophilic compounds, ABCC1/MRP1 can transport lipophilic anions like leukotriene C4, glucuronate conjugates and sulfated bile acids. In addition, ABCC1/MRP1 takes GSH-conjugates as substrates, a property it shares with most other C subfamily members (MRPs) (Ballatori et al. 2009). ABCC1/MRP1 is widely expressed in various tissues, including the respiratory system and gastrointestinal tract (as main entry pathways for entry of Cd and Cd-complexes), testis, kidney, heart, placenta, and to a lesser extent in the liver (Bakos and Homolya 2007; https://www.proteinatlas.org/ENSG00000103222-ABCC1/tissue). Moreover, contrary to ABCB1/Pgp, ABCC1 is expressed in the basolateral membrane in polarized epithelial cells.

#### Cd and ABCC1

The ABC transporter yeast cadmium resistance factor 1 (YCF1), the yeast ortholog of mammalian ABCC1 and ABCC2, mediates transport of GSH and bis(glutathionato)Cd, thereby conferring Cd resistance by its elimination from the cytosol and intracellular compartmentation into the yeast vacuole (Li et al. 1997). Human ABCC1/MRP1 can rescue Cd transport activity in a *YCF1* deletion strain (Tommasini et al. 1996). Therefore, it is very likely that ABCC1/MRP1 is an efflux pump for Cd-GSH complexes.

This hypothesis is supported by the observation that inhibition of Abcc1 by MK571 results in increased tissue accumulation of Cd in zebrafish exposed to low micromolar Cd concentrations, as does *Abcc1* knockout (Tian et al. 2017). Interestingly, ABCC1/MRP1 (as well as ABCC2) has been proven to transport arsenite (As^3+^) as a triglutathione conjugate, and with an apparent *K*_*m*_ of 0.32 μM (Leslie et al. 2004). Recently, Grau-Perez et al. (Grau-Perez et al. 2021) investigated genetic determinants of urine Cd in American Indian adults. They found strong statistical evidence for a genetic locus at chromosome 16 determining urine Cd concentrations. Among the top 20 associated SNPs in this locus, 17 were annotated to *ABCC1,* supporting that urinary Cd levels are heritable and influenced by a quantitative trait locus (QTL) linkage on chromosome 16, which may be explained by genetic variation in *ABCC1*. Yet, it would be useful to directly show that ABCC1/MRP1 transports Cd, e.g. by radioactive tracer studies.

### ABCC7 (CFTR/cystic fibrosis transmembrane conductance regulator)

Based on its structure, function and regulation, CFTR is an ABC transporter (ABCC7) (Liu et al. 2017); reviewed in (Csanady et al. 2019; Thomas and Tampe 2020)). However, ABCC7/CFTR is a unique ABC transporter because it functions as a low conductance Cl^−^-selective channel gated by cycles of ATP binding and hydrolysis at its NBDs, and conducting Cl^−^ anions down their electrochemical gradient, whereas most ABC transporters transport their substrates against a chemical gradient under ATP hydrolysis. ABCC7/CFTR-mediated anion flow is needed for normal function of secretory epithelia and the channel protein is located primarily in the apical membrane of polarized epithelial cells (Crawford et al. 1991; Denning et al. 1992), such as those lining airways, the intestinal tract, pancreas, bile ducts, testes, sweat glands and kidney (Gadsby et al. 2006). ABCC7/CFTR is tightly regulated by an intrinsically disordered protein segment, which contains multiple consensus phosphorylation sites and is termed the regulatory domain (Bozoky et al. 2013). Mutations in the ABCC7/CFTR gene cause cystic fibrosis (CF), the most common fatal hereditary lung disease among people of Northern European ancestry (Wang et al. 2014).

#### Cd and ABCC7/CFTR

Cd affects gating and inhibits ATP hydrolysis of ABCC7/CFTR without being transported (Annereau et al. 2003; El Hiani and Linsdell 2014). Similar to other members of the ABC protein family, ABCC7/CFTR mediates GSH export from cells (Kogan et al. 2003; Linsdell and Hanrahan 1998). GSH is the major antioxidant in the extracellular lining fluid of the lung; consequently, reduced GSH transport due to ABCC7/CFTR mutations may contribute to the pathology of CF (Roum et al. 1993). Accordingly, the prooxidant Cd and antioxidant GSH have opposite effects on redox signaling and affect ABCC7/CFTR channel gating accordingly (Harrington et al. 1999). Concerning possible Cd transport function of ABCC7/CFTR, low micromolar concentrations of Cd trigger ABCC7/CFTR-like Cl^−^ currents and ABCC7/CFTR-mediated GSH efflux in primary renal PT cells (L’Hoste et al. 2009). Once activated, ABCC7/CFTR appears to transport GSH and Cd-GSH conjugates because the authors showed that ABCC7/CFTR is directly involved in the efflux of Cd by comparing Cd efflux in *cftr*^+*/*+^ and *cftr*^*−/−*^ cultured cells, and the Cd efflux rate is correlated to the intracellular concentration of GSH. In PT cells, the increase of GSH permeation through ABCC7/CFTR could have at least two consequences: (1) direct exit of Cd-GSH conjugates that could contribute to a rapid detoxification of PT cells by pumping out cytosolic Cd; (2) depletion of GSH that decreases the capacity of the cell to scavenge ROS produced by free Cd. Based upon their findings, the authors hypothesized that ABCC7/CFTR may extrude Cd-GSH as previously described in the case of the yeast cadmium resistance factor YCF1 (Li et al. 1997). However, it should be emphasized that L’Hoste et al. (L’Hoste et al. 2009) did not provide direct experimental evidence for ABCC7/CFTR-mediated Cd-GSH transport. Sequence comparison of selected ABC transporters, such as ABCB1/Pgp, ABCC1/MRP1, ABCC2, ABCC7/CFTR, and YCF1 proteins suggested that CFTR/MDR-like proteins (ABCB1/Pgp, ABCC7/CFTR) lack an N-terminal hydrophobic membrane-bound domain (TMD0) and a “lasso” motif (L0 linker) that both may be crucial for transport of typical organic anionic substrates, such as GSH (Tusnady et al. 1997). However, subsequent work did not confirm a role for TMD0 in GSH transport by ABCC1/MRP1 (Bakos et al. 1998). However, ABCC7/CFTR displays a L0 lasso motif that resembles that of ABCC1/MRP1 and could play a role in GSH transport and Cd resistance (Mason and Michaelis 2002).

### ABCG2 (BCRP/breast cancer resistance protein)

ABCG2/BCRP is highly expressed in the luminal membrane of the intestine and renal tubule, endothelial cells of the blood-brain-barrier, smooth muscle cells and reproductive tissue, in particular in syncytiotrophoblasts, the transporting epithelium of the placenta, and glandular cells of the seminal vesicle. Furthermore, together with ABCB1/Pgp and ABCC1/MRP1, it is widely reported to be upregulated in multidrug resistant cancers from various tissue origins (Litman et al. 2000; Natarajan et al. 2012), with focus on breast cancer in which it was discovered (Modi et al. 2022; van der Noord et al. 2023). The spectrum of substrates transported by ABCG2/BCRP is very broad and overlaps substantially with, yet is distinct from, ABCB1/Pgp and ABCC1/MRP1. ABCG2/BCRP favors organic anions, including sulfated GSH and glucuronide conjugates. It also transports several chemotherapeutics and tyrosine kinase inhibitors (Mao and Unadkat 2015).

#### Cd and ABCG2/BCRP

With abundant ABCG2/BCRP expression, the placenta has been the major focus of Cd in relation to this ABC transporter. Forming a filter, safety barrier as well as the site of nutrient/waste/signaling exchange, the syncytiotrophoblasts have to be well-equipped to perform these functions. Indeed, 11 members of the ABC transporter family and 9 members of the solute carrier (SLC) family have been confirmed in this tissue (Walker et al. 2017; Taggi et al. 2022). In a study employing quantitative targeted proteomics, ABCG2/BCRP and ABCB1/Pgp were the most abundant ABC transporters in human placental membranes with highest expression in the first trimester and decreasing by 55 and 69%, respectively, at full term (Anoshchenko et al. 2020). Analogous to the renal PT, the plethora of transporters makes the placenta susceptible to metal toxicity as there are many ways to cross the cell membrane. Accumulation of Cd in the placenta compared to the urine and umbilical cord blood has been reported in both rodents and humans (Kippler et al. 2010; Jacobo-Estrada et al. 2017; Laine et al. 2015; Piasek et al. 2014) and can affect the uptake of essential ions, such as zinc (Kippler et al. 2010). Placental ABCG2/BCRP seems to be negatively affected by Cd, which reduces functional activity, though not necessary in parallel with attenuated expression (Kummu et al. 2012; Liu et al. 2016). Moreover, infants harboring the reduced function ABCG2/BCRP polymorphism C421A (Q141K) suffered from retarded fetal development, smaller placentas and increased placental Cd (Barrett et al. 2023). In ABCG2/BCRP-overexpressing HEK293 cells treated with 0.5 µM CdCl_2_ for 30 min and incubated for 60 min after Cd washout, less Cd was accumulated compared to empty vector.  In support, kidneys from *Abcg2*^*−/−*^ mice injected with 5.5 mg/kg CdCl_2_, i.p., Cd accumulation was augmented, suggesting Cd is transported by ABCG2/BCRP (Wen et al. 2021). Moreover, oxidative stress markers and consequent apoptotic cell death induced by Cd were reduced in ABCG2/BCRP-expressing HEK293 cells compared to empty vector or ABCG2/BCRP-Q141K transfected cells (Wen et al. 2021). The speciation of the Cd that is hypothetically transported is not known. Previous transport studies imply GSH is not a substrate of ABCG2/BCRP (Gauthier et al. 2013) and more recent structural studies point to ABCG2/BCRP transporting substrates that are generally polycyclic and hydrophobic in nature proffered by two cholesterol molecules bound in the substrate pocket (Taylor et al. 2017; Yu et al. 2021), which do not reconcile with the chemistry of Cd. Alterations of ABCG2/BCRP may impact the cellular response to Cd and/or the expression of other transporters involved in Cd uptake and efflux, though Oct2/*Slc22a2*, Dmt1/*Slc11a2*, Zip8/*Slc39a8*, Zip14/*Slc39a14*, Mate1/*Slc47a1* and *Abcb1a/b* transcripts were not affected in *Abcg2*^*−/−*^ mice (Wen et al. 2021). An alternative explanation is the transport of an as-yet-unidentified substrate, which binds or affects Cd accumulation.

### Other ABC transporters

In placental tissue of Cd-treated rats, ABCB4/MRP3 was identified as a Cd target by differential gel electrophoresis (DIGE) combined with matrix-assisted laser desorption/ionization time-of-flight tandem mass spectroscopy (MALDI-TOF/TOF MS). Downregulation of ABCB4 by Cd was confirmed by immunoblotting (Liu et al. 2016). Little is known about ABCB4, though it has been implicated in cancer progression and multidrug resistance as well as in phospholipid transport in the liver. Mutations in ABCB4 result in diseases associated with defective gall bladder function and reduced bile flow (cholestasis) (Sticova and Jirsa 2020).

Interestingly, Cd-resistant fibroblast-like zebrafish (ZF4-Cd) cells show increased expression of *abcc2* and *abcc4* (but not *abcc1*) and exhibit decreased Cd content, enhanced MK571-dependent accumulation of MRP substrates calcein-AM and rhodamine 123 along with increased cellular GSH compared to Cd-sensitive cells as well as cross-resistance to mercury, arsenite and arsenate. This suggests that ABCC2/4 transporters are involved in the efflux of Cd conjugated with cellular GSH and thus play crucial roles in Cd detoxification of zebrafish cells (Long et al. 2011). In a subsequent study, functional expression of ABCC2/MRP2 and ABCC1/MRP1 was studied in zebrafish embryos at time points between 4 and 72 h post-fertilization, which increased with time and correlated with an increased tolerance to the toxicity caused by CdCl_2_. Moreover, MK571 significantly inhibited the efflux (as measured by atomic absorption spectrometry of Cd in the lysates) of Cd and increased its toxicity in zebrafish embryos (Yin et al. 2016).

## Concluding remarks and outlook

**Fig. 2:**
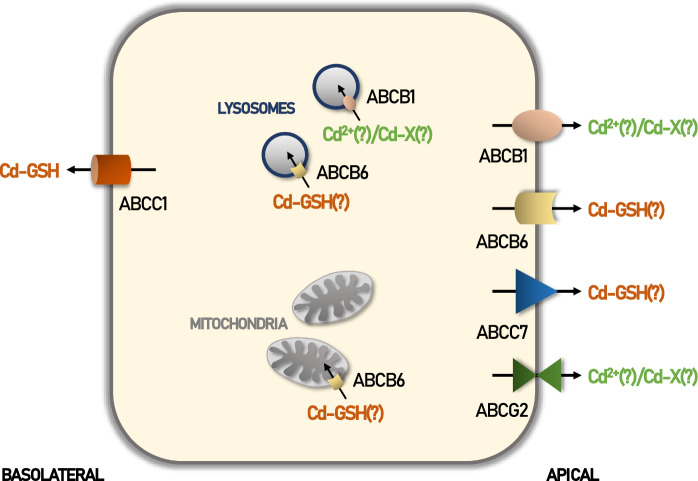
Synopsis of cadmium-transporting ABC transporters. Vectorial transport of cadmium ions (Cd^2+^), cadmium bound to glutathione (Cd-GSH) or yet unidentified cadmium-compounds (Cd-X) has been reported for ABCB1/Pgp, ABCB6, ABCC7/CFTR and ABCG2/BCRP in the luminal membrane of epithelial cells. ABCC1 transports Cd-GSH across the basolateral membrane. Intracellular ABCB1/Pgp and ABCB6 have been localized to mitochondria and/or lysosomes where they could also potentially mediate transport of Cd^2+^ and Cd-GSH into the organelles. “(?)” indicates a postulated mechanism without definitive proof. Please refer to text for further details and references

With the exception of ABCB6 (Rakvacs et al. 2019) and ABCC1/MRP1 (Tommasini et al. 1996; Tian et al. 2017) (see Fig. 2), only weak evidence for efflux of Cd by mammalian ABC transporters exists. Hence, further research is required to clarify Cd transport by other ABC transporters and the transported chemical form, i.e. either as free ion or complexed to organic substrates of these ABC transporters (Fig. 2).

It is striking that with both ABCB6 and ABCC1/MRP1, Cd efflux may only occur when it forms a complex with their physiological substrate, the tripeptide GSH (see “Cd and ABCB6” and “Cd and ABCC1”). Thus, this could represent a strategy for Cd detoxification. Indeed, this concept is not novel (Singhal et al. 1987), but has not been systematically investigated in vivo. The mechanism of detoxification may hence involve ABC transporter-mediated efflux of Cd-GSH complexes. Consequently, GSH synthesis could be promoted by various means, including addition of N-acetylcysteine (NAC) (Yim et al. 1994; Whillier et al. 2009). Indeed, sporadic studies have confirmed the practicability of this therapeutic approach (Gil et al. 2011).

However, two caveats need to be considered: (1) Cd interferes with enzymes involved in the GSH antioxidant system (reviewed in Waisberg et al. 2003; Thévenod 2009) and (2) this detoxification strategy could preferentially be useful for acute Cd intoxication, as GSH synthesis from NAC is rapid and occurs within minutes (Whillier et al. 2009). Moreover, a problem with chronic Cd intoxication is the induction of Cd-chelating MT within cells, which leads to accumulation of potentially toxic high intracellular Cd concentrations (Sabolic et al. 2010). The affinity of MT to Cd is significantly higher (~ 10^-14^ M) (reviewed in (Romero-Isart and Vasak 2002) than that of GSH (~ 10^-10^ M) (Perrin and Watt 1971), which could avert removal of Cd from cells via complexation with GSH and ABC transporter-mediated efflux.

An interesting clinically relevant approach is treatment of acute Cd intoxication in rats with water-dimercaprol (British anti-Lewisite) (Bernhoft 2013), the Cd chelating agent 2, 3-dimercapto-1-propane sulfonic acid (DMPS), in combination with cysteine or NAC, which improves Cd mobilization from extrarenal tissues (e.g. liver) (Tandon et al. 2002) compared to DMPS alone, suggesting that ABC transporter-mediated Cd efflux could contribute to Cd removal from those tissues by combined treatment. Unfortunately, Cd removal from the kidneys was not improved, indicating that Cd mobilized from other body sites was redistributed to the kidneys.

Another potentially clinically relevant issue is the observation that the *ABCG2* genetic variant Q141K exhibits altered membrane trafficking, which results in reduced efflux of ABCG2/BCRP substrates, including Cd (see “Cd and ABCG2/BCRP” and (Wen et al. 2021). Individuals exhibiting the *ABCG2* genetic variant Q141K could be more sensitive to chronic exposure to Cd by food, tobacco smoking and environmental pollution and hence Cd toxicity of the kidneys and other organs (including intestinal tract, brain, and placenta).

Nevertheless, it remains mandatory to elucidate other mechanisms of Cd efflux by ABC transporters, either as free Cd or as Cd complexed to other yet unknown molecules (see Fig. 2), for detoxification purposes.

## Data Availability

The data are available in the cited publications.
